# National survey on the current practice and attitudes toward the management of chronic subdural hematoma

**DOI:** 10.1002/brb3.2463

**Published:** 2022-02-03

**Authors:** Dana C. Holl, Jurre Blaauw, Erwin Ista, Clemens M.F. Dirven, Kuan H. Kho, Korné Jellema, Niels A. van der Gaag, Ishita P. Miah, Heleen M. den Hertog, Joukje van der Naalt, Bram Jacobs, Dagmar Verbaan, Suzanne Polinder, Hester F. Lingsma, Ruben Dammers, Dutch Subdural Hematoma Research group collaboration

**Affiliations:** ^1^ Department of Neurosurgery Erasmus Medical Center, Erasmus MC Stroke Center Rotterdam the Netherlands; ^2^ Department of Public Health Erasmus Medical Center Rotterdam the Netherlands; ^3^ Department of Neurology University Groningen, University Medical Center Groningen Groningen the Netherlands; ^4^ Department of Internal Medicine Nursing Science, Erasmus Medical Center Rotterdam the Netherlands; ^5^ Department of Neurosurgery Medisch Spectrum Twente Enschede the Netherlands; ^6^ Department of Neurology Haaglanden Medical Center Hague the Netherlands; ^7^ Haaglanden Medical Center, Haga Teaching Hospital University Neurosurgical Center Holland (UNCH) Leiden University Medical Center Leiden the Netherlands; ^8^ Department of Neurology Amphia Hospital Breda the Netherlands; ^9^ Department of Neurology Isala Hospital Zwolle Zwolle the Netherlands; ^10^ Department of Neurosurgery, Neurosurgical Center Amsterdam Amsterdam University Medical Centers Amsterdam the Netherlands

**Keywords:** chronic subdural hematoma, guideline, surveys and questionnaires, traumatic brain injury, treatment

## Abstract

**Background:**

Chronic subdural hematoma (CSDH) is a frequent pathological entity in daily clinical practice. However, evidence‐based CSDH‐guidelines are lacking and level I evidence from randomized clinical trials (RCTs) is limited. In order to establish and subsequently implement a guideline, insight into current clinical practice and attitudes toward CSDH‐treatment is required. The aim is to explore current practice and attitudes toward CSDH‐management in the Netherlands.

**Methods:**

A national online survey was distributed among Dutch neurologists and neurosurgeons, examining variation in current CSDH‐management through questions on treatment options, (peri)operative management, willingness to adopt new treatments and by presenting four CSDH‐cases.

**Results:**

One hundred nineteen full responses were received (8% of neurologists, *N* = 66 and 35% of neurosurgeons, *N* = 53). A majority of the respondents had a positive experience with burr‐hole craniostomy (93%) and with a conservative policy (56%). Around a third had a positive experience with the use of dexamethasone as primary (30%) and additional (33.6%) treatment. These numbers were also reflected in the treatment preferences in the presented cases. (Peri)operative management corresponded among responding neurosurgeons. Most respondents would be willing to implement dexamethasone (98%) if equally effective as surgery and tranexamic acid (93%) if effective in CSDH‐management.

**Conclusion:**

Variation was found regarding preferential CSDH‐treatment. However, this is considered not to be insurmountable when implementing evidence‐based treatments. This baseline inventory on current clinical practice and current attitudes toward CSDH‐treatment is a stepping‐stone in the eventual development and implementation of a national guideline.

## 1 INTRODUCTION

Chronic subdural hematoma (CSDH) is a collection of blood, blood breakdown products, and cerebrospinal fluid in the subdural space, which occurs frequently in neurological and neurosurgical practice. Worldwide, burr‐hole craniostomy is the mainstay in CSDH‐management (Kolias et al., 2014; Soleman et al., 2017). The perioperative placement of a subdural drain was the first level I evidence available in CSDH research, associated with a reduced recurrence rate and mortality at 6 months (Santarius et al., 2009). However, recent publications suggested that a subperiosteal drain leads to fewer recurrences compared to a subdural drain ( Greuter et al., 2020; Pranata et al., 2020; Soleman et al., 2019). Using a drain is beneficial, but the exact definition and way of using these two types of drains are still part of the debate on CSDH management. There is an ongoing lack of evidence‐based guidelines regarding the optimal treatment for the individual patient diagnosed with CSDH. As a result, there is wide variability in the treatment of this condition. This variability is not only seen internationally, but also at a national, regional, and interhospital level, and even among treating physicians (Kolias et al., 2014). Therefore, there is an ongoing need for evidence‐based guidelines in CSDH. Especially because CSDH mainly affects elderly patients. The incidence of CSDH is expected to rise because of the large ageing population (United Nations) and the increasing use of antiplatelet agents and anticoagulants (Vacca & Argento, 2018). Therefore, complications and side effects of (non)surgical treatments must be carefully considered when creating a guideline for this fragile population.

Before establishing and implementing a guideline, barriers that can cause nonadherence to guidelines should be identified. Nonadherence to guidelines can be caused by a wide range of barriers, most prominently a lack of agreement with guideline recommendations, but also because of environmental barriers such as organizational constraints and a lack of collaboration (Lugtenberg et al., 2009). Implementation of guidelines can also be complicated by a lack of awareness of the existence of a guideline, lack of familiarity with the guideline or with the advised treatment, lack of outcome expectancy, and the inertia of previous practice (Cabana et al., 1999).

To detect and explore these barriers, which can be faced when implementing a guideline, three steps should be considered. First, it is important to understand the underlying pathophysiological mechanism of CSDH to understand why a proposed treatment should be effective. The development of CSDH relies on a complex intertwined pathway of angiogenesis, inflammation, recurrent small bleeds from immature capillaries, exudates, and local coagulopathy (Edlmann et al., 2017; Holl et al., 2018). Second, to prevent a lack of agreement with recommendations, it is essential to provide level I evidence on CSDH treatment through high‐quality RCTs. Twenty‐six RCTs are currently running or have recently been published on various treatment options, among which steroids, tranexamic acid, statins, surgical techniques, middle meningeal artery embolization, and perioperative care (Edlmann et al., 2020). The third step is to explore the current clinical practice and current attitudes toward CSDH‐management among treating physicians. Through this exploration, one can study the outcome expectancy of these treatments and detect familiarity with possible treatments, with possible environmental barriers, and with the inertia of current practice. If major resistance or large differences among treating physicians are found, this might be a barrier when implementing evidence‐based treatments.

We conducted an online survey among Dutch neurologists and neurosurgeons. In the Netherlands, neurologists usually diagnose CSDH. Hereafter, the patient can be treated with an expectant policy, with a nonsurgical treatment (e.g., dexamethasone), or through surgery. If necessary, the neurologist consults a neurosurgeon on further management. They will reach consensus and, if an operation is the preferred option, will transfer the patient to the nearest neurosurgical center. In the end, these two specialisms will decide on the best treatment option for each individual CSDH‐patient. Therefore, only these two specialisms were invited to complete this survey, which aims to explore current practice and attitudes toward CSDH‐management in The Netherlands.

## 2 METHODS

We conducted a national online survey to explore the current experience with different treatment options for CSDH among the total population of Dutch neurologists (*N* = 844) and Dutch neurosurgeons (*N* = 153). The finalized version was converted into an online questionnaire, using a data management open‐source software program (LimeSurvey, version 2.06LTS). The link of the survey was spread among neurologists through the website, newsletter, and LinkedIn page of the Netherlands Society of Neurology and among neurosurgeons by two emails through the Dutch Neurosurgical Society. The link to the online survey was also spread by members of the Dutch Subdural Hematoma Research group (DSHR) among their direct contacts within neurology and neurosurgery departments in several Dutch hospitals.

### 2.1 Survey

The online survey was developed by two of the authors (D.C.H. and J.B.) with input from an implementation specialist (E.I.). The survey was revised by the DSHR members and comments were incorporated in a pilot version that was completed by 10 DSHR members. The process of revision by DSHR members was repeated until consensus on the final questionnaire was reached. Research reporting guidelines were consulted, but none of the EQUATOR guidelines matched the design of this survey. No ethical approval was obtained for this low‐risk survey. The survey was spread among colleagues in neurology and neurosurgery; no patients were involved in this research. Data were collected voluntarily and anonymously and participants could not be identified from the results.

The survey consisted of five domains: (1) demographics and other respondents' characteristics; (2) opinion on different treatment strategies; (3) treatment choices in four separate CSDH cases; (4) willingness to implement Dutch CSDH RCT results; (5a) question on CSDH‐treatment for neurologists only, (5b) treatment questions for neurosurgeons only. The exploration of these five domains enabled us to evaluate similarities and variations in current clinical practice and attitudes toward CSDH‐management.

In domain 2, “opinion of different treatment strategies,” the opinion on six treatment options was explored: (1) the use of (additional) dexamethasone, (2) (additional) tranexamic acid and (3) (additional) statins, (4) the performance of middle meningeal artery embolization, (5) burr hole craniostomy (BHC), and (6) the choice for a conservative policy in which no surgical nor medical management is performed.

In domain 3, “treatment choices in four separate cases” were explored. These four cases of CSDH were presented to the respondents. Each case represents the same patient with the same underlying cause of the CSDH, but with different outcomes on CT‐imaging and different neurological exams.

In domain 4, we looked into the “willingness to implement Dutch CSDH RCT results.” This concerns the willingness to adopt a theoretically potential positive outcome of two prospective multicenter RCTs: the DECSA‐trial and the TORCH‐trial. The DECSA trial (DExamethasone therapy in symptomatic patients with Chronic Subdural hematomA) aimed to evaluate the effect of initial dexamethasone therapy versus primary surgery on functional outcome and cost‐effectiveness in symptomatic CSDH‐patients (Miah et al., 2018). This RCT was recently terminated and results are awaited. The TORCH‐trial (Tranexamic acid to prevent OpeRation in Chronic subdural Hematoma) is a double‐blind, placebo‐controlled trial that aims to evaluate the efficacy of TXA to prevent surgery in CSDH‐patients for whom conservative treatment is selected as a primary treatment strategy (Clinicaltrials.gov). Domain 5 involves questions on current clinical practice. We explored to what extent treatment techniques of responding neurosurgeons correspond. Organizational constraints were evaluated by questioning operation room capacity. Collaboration, or a lack thereof, was explored by asking neurologists if and when they would consult a neurosurgeon. These examples were explored through different questions in order to identify possible environmental barriers.

### 2.2 Analysis

Data were collected from 19 May 2020 to 4 November 2020. All data retrieved through this survey were entered into and analyzed with SPSS 25.0 (SPSS®, Chicago, Il, USA). Standard descriptive statistics were used. A minimal statistical level of accuracy of 15% with a 95% confidence level was considered to be acceptable (van Bennekom, 2002). We calculated the statistical accuracy of this survey in both groups using the population size in combination with the percentage of responding neurologists and neurosurgeons. A higher percentage of responses naturally leads to higher accuracy. Also, a larger population size needs fewer responses to achieve the same statistical accuracy as in a smaller population; statistical accuracy and response rate are not linear (Van Bennekom, 2002; Van Bennekom, 2021). Results were described for neurologists and neurosurgeons separately.

## 3 RESULTS

**FIGURE 1:**
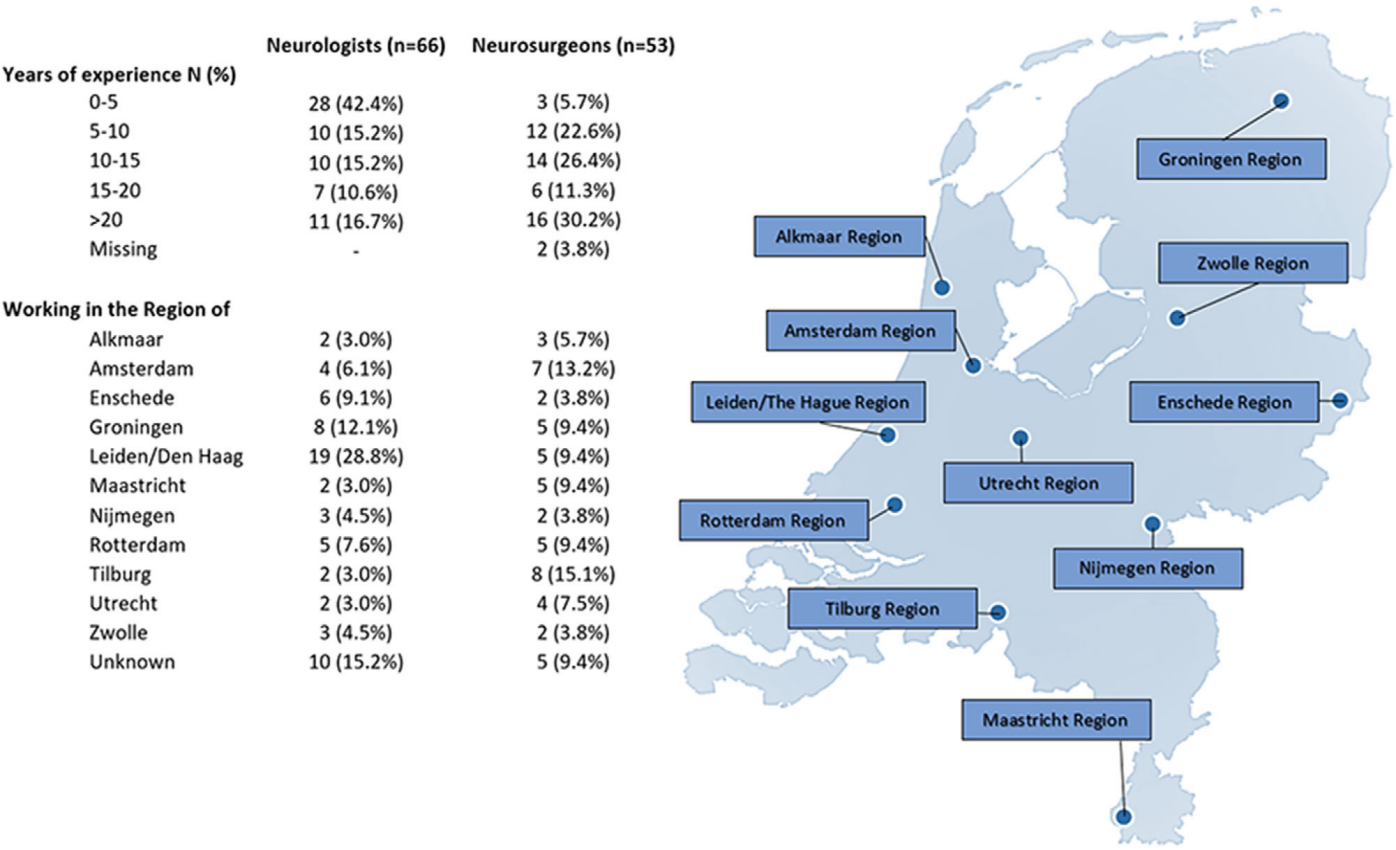
Demographics of respondents to a national survey examining views of Dutch neurologists and neurosurgeons on current practice and attitudes toward the management of chronic subdural hematoma

A total of 123 responses were received of which 119 full responses from 31 Dutch hospitals (see Figure 1).

The four partial responses were removed from the results. The survey was fully completed by 12% (119/997) of Dutch neurologists and neurosurgeons approached. These responses are divided into a response rate of 8% (66/844) among neurologists and 35% (53/153) among neurosurgeons. With a response of 8% within a population of 844, the statistical level of accuracy in the neurological survey is ±12% with a 95% confidence level. With a response of 35% within a population of 153, the statistical level of accuracy in the neurosurgical survey is ±11% with a 95% confidence level (van Bennekom, 2002). Responses received from neurologists and neurosurgeons were largely consistent. Therefore, the differences between the groups were not explicitly included in the written results. These differences between responding neurologists and neurosurgeons can be found in the tables and figures.

### 3.1 Demographics and other respondents' characteristics

A 0–5 years’ work experience was seen in 28 (42%) of the responding neurologists and three (6%) of the responding neurosurgeons. A work experience of 20 years or more was seen in 11 (17%) of the responding neurologists and 16 (30%) of neurosurgeons (see Figure 1). No major differences were found related to the years of work experience. Therefore, this has no impact on the presented results.

### 3.2 Opinion on different treatment strategies

A substantial majority of respondents were positive on BHC as CSDH treatment (93.3%); none of the respondents had a negative experience with BHC. More than half of the respondents were positive about a conservative policy in CSDH (56.3%). Around a third of respondents had a positive experience with the use of dexamethasone as primary (30.3%) and additional (33.6%) treatment. Less than 10% had a negative experience with the use of dexamethasone. The vast majority of respondents had (almost) no experience with the use of tranexamic acid, statins, and middle meningeal artery embolization (see Table 1).

**TABLE 1:** Opinion of Dutch neurologists and neurosurgeons on different treatment strategies

		Experience			
What is your experience with…	Specialism	(Almost) none	Good	Bad	Neutral
**…primary DXM**	Neurology (*N* = 66)	21	19	8	18
	Neurosurgery (*N* = 53)	16	17	2	18
	**Total (*N* = 119)**	**37 (31.1%)**	**36 (30.3%)**	**10 (8.4%)**	**36 (30.3%)**
**…additional DXM**	Neurology (*N* = 66)	36	15	3	12
	Neurosurgery (*N* = 53)	20	25	3	5
	**Total (*N* = 119)**	**56 (47.1%)**	**40 (33.6%)**	**6 (5.0%)**	**17 (14.3%)**
**…primary TXA**	Neurology (*N* = 66)	61	1	2	2
	Neurosurgery (*N* = 53)	41	5	1	6
	**Total (*N* = 119)**	**102 (85.7%)**	**6 (5.0%)**	**3 (2.5%)**	**8 (6.7%)**
**…additional TXA**	Neurology (*N* = 66)	62	1	1	2
	Neurosurgery (*N* = 53)	43	4	2	4
	**Total (*N* = 119)**	**105 (88.2%)**	**5 (4.2%)**	**3 (2.5%)**	**6 (5.0%)**
**…primary statins**	Neurology (*N* = 66)	62	0	1	3
	Neurosurgery (*N* = 53)	53	0	0	0
	**Total (*N* = 119)**	**115 (96.6%)**	**(0.0%)**	**1 (0.8%)**	**3 (2.5%)**
**…additional statins**	Neurology (*N* = 66)	63	0	1	2
	Neurosurgery (*N* = 53)	53	0	0	0
	**Total (*N* = 119)**	**116 (97.5%)**	**(0.0%)**	**1 (0.8%)**	**2 (1.7%)**
**…MMA^§^ embolization**	Neurology (*N* = 64)	62	0	1	1
	Neurosurgery (*N* = 53)	50	0	0	3
	**Total (*N* = 117)**	**112 (95.7%)**	**(0.0%)**	**1 (0.9%)**	**4 (3.4%)**
**…burr‐hole craniostomy**	Neurology (*N* = 66)	2	58	0	6
	Neurosurgery (*N* = 53)	0	53	0	0
	**Total (*N* = 119)**	**2 (1.7%)**	**111 (93.3%)**	**(0.0%)**	**6 (5.0%)**
**…expectative/conservative therapy**	Neurology (*N* = 66)	0	40	6	20
	Neurosurgery (*N* = 53)	3	27	7	16
	**Total (*N* = 119)**	**3 (2.5%)**	**67 (56.3%)**	**13 (10.9%)**	**36 (30.3%)**

DXM = dexamethasone; TXA = tranexamic acid; MMA = middle meningeal artery.

In order to assess the collaboration between neurologists and neurosurgeons, we evaluated the communication or a lack thereof between these specialisms. The majority of responding neurologists indicated they always consult with a neurosurgeon when a CSDH patient has neurological deficits. A third of neurologists stated that the decision to consult a neurosurgeon depends on the severity of CT scan results. Twenty‐two percent of neurologists stated they always consult with a neurosurgeon when they have diagnosed patient with CSDH (Table 2).

**TABLE 2:** When would a responding neurologist consult a neurosurgeon concerning CSDH‐treatment?

Dutch neurologist: “I would consult a neurosurgeon…”	
…in case of neurological deficit(s)	36/58 (62.1%)
…based on brain imaging	20/58 (34.5%)
…(almost) always	13/58 (22.4%)
…*n* case of decreased consciousness	10/58 (17.2%)
…when in doubt	2/58 (3.4%)
…if anticoagulants/antithrombotics are used	1/58 (1.7%)
No answer provided	8/66 (12.1%)

### 3.3 Treatment choices in four separate CSDH cases

Multiple answers were possible in the presented cases.

**FIGURE 2:**
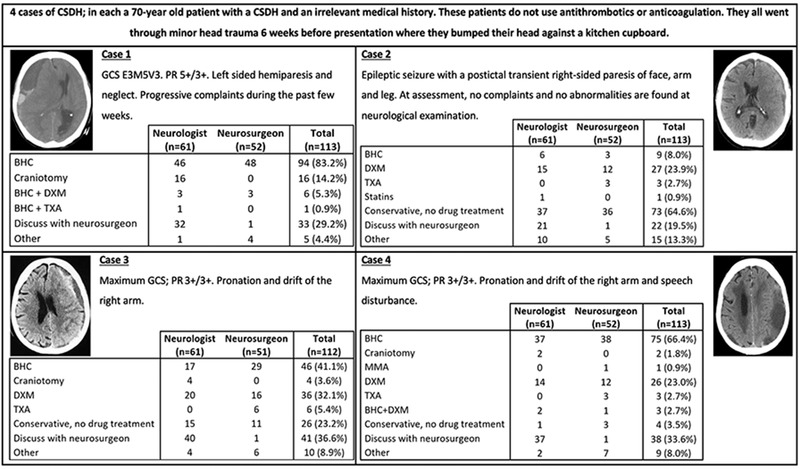
Treatment choices in four fictitious CSDH cases which were presented to the respondents of this national survey. GCS = Glasgow Coma Scale; PR = pupillary reflex; BHC = burr‐hole craniostomy; DXM = dexamethasone; TXA = tranexamic acid; MMA = middle meningeal artery embolization

In case 1, a vast majority of 83% chose primary BHC. In addition, primary craniotomy was opted by 16 (26%) neurologists and none of the neurosurgeons (see Figure 2).

In case 2, a majority of 65% chose an expectant policy. The second‐largest option with 24% was primary dexamethasone. In addition, 13% of respondents opted for “other” treatment with (additional) antiepileptic drugs (see Figure 2).

In case 3, a majority of 41% chose primary BHC. With 32%, the second‐largest option was primary dexamethasone. Twenty‐three percent of respondents chose an expectant policy (see Figure 2).

In case 4, a majority of 66% chose BHC. The second‐largest option with 23% is primary dexamethasone (see Figure 2).

Overall, 20–37% of respondents reasoned the case should be discussed with a neurosurgeon before starting a particular treatment.

### 3.4 Willingness to implement Dutch CSDH RCT results

**FIGURE 3:**
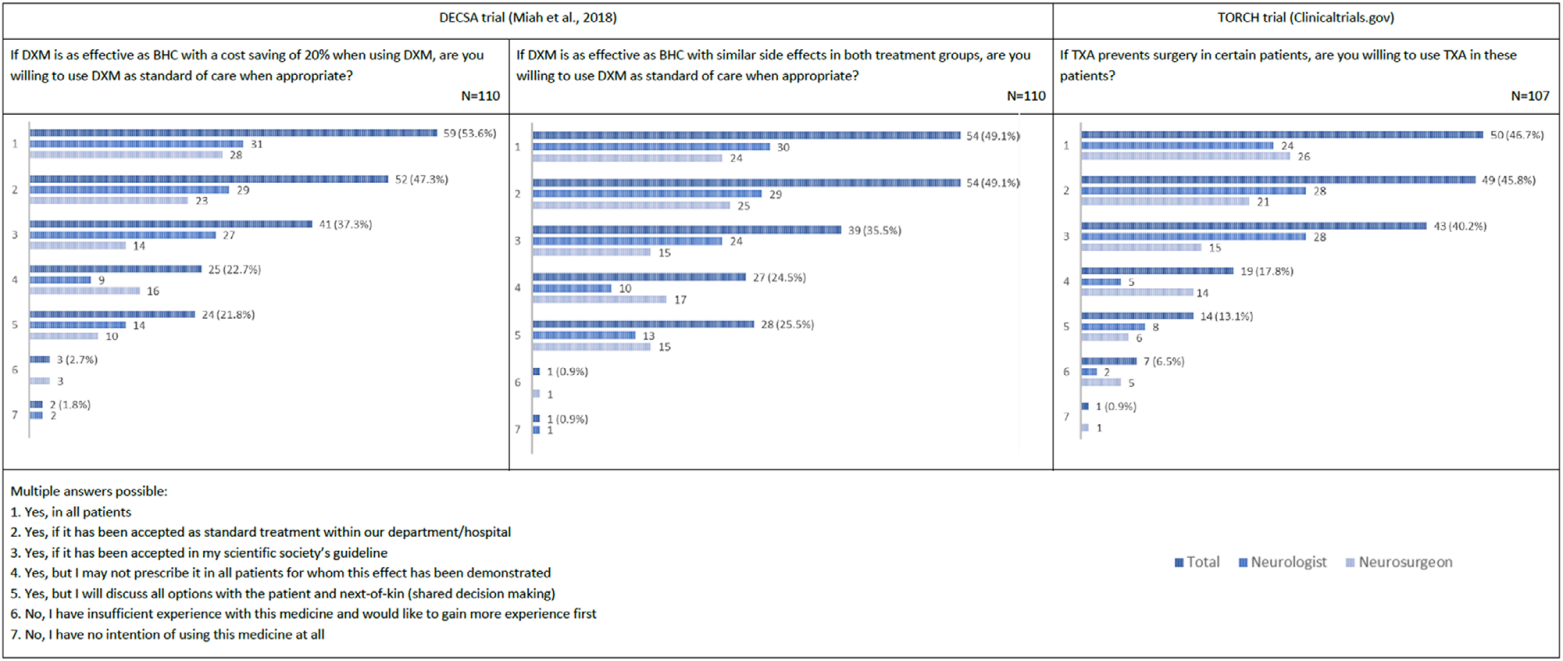
Willingness among Dutch neurologists and neurosurgeons to implement Dutch CSDH RCT results. DXM = dexamethasone; BHC = burr‐hole craniostomy; TXA = tranexamic acid

If the DECSA trial demonstrates that dexamethasone is equally effective or cost‐effective compared to BHC, the majority of respondents would be willing to implement the use of dexamethasone (see Figure 3). Also, the majority of respondents would be willing to use tranexamic acid as the standard of care if a positive effect is shown through the TORCH trial (see Figure 3).

### 3.5 Questions on current clinical practice

The different operative techniques largely correspond among responding neurosurgeons (see Table 3).

**TABLE 3:** Preferred (peri)operative treatment techniques of responding Dutch neurosurgeons

**Which type of anesthesia do you most often use?**	Local	8/47 (17.0%)
	General	39/47 (83.0%)
	Missing	6
**Which amount of burr‐holes do you most often use?**	1	32/47 (68.1%)
	2	15/47 (31.9%)
	>2	(0%)
	Missing	6
**In case of a bilateral CSDH the patient is operated on…**	…the symptomatic side only	14/47 (29.8%)
	…both sides if technically possible	33/47 (70.2%)
	Missing	6
**Do you flush the subdural space in general?**	No	2/49 (4.1%)
	Yes	47/49 (95.9%)
	*Flush on room temperature*	*10/47 (21.3%)*
	*Flush on body temperature*	*37/47 (78.7%)*
	Missing	4
**If you flush, which sort of fluid do you use to flush the subdural space?**	Sterofundin ISO solution	6/46 (13.0%)
	Ringer's lactate solution	12/46 (26.1%)
	Physiological salt solution	27/46 (58.7%)
	Unknown	1/46 (2.2%)
	Missing	7
**Do you usually use burr‐hole covers?**	Yes	(0%)
	No, but I am willing to	16/48 (33.3%)
	No, this is not necessary in my opinion	27/48 (56.3%)
	No, the advantages do not outweigh the disadvantages and risks	5/48 (10.4%)
	Missing	5
**Do you usually place a drain?**	No	1/46 (2.2%)
	Yes	46/47 (97.9%)
	*Subgaleal*	*11/46 (23.9%)*
	*Subdural*	*35/46 (76.1%)*
	Missing	6
**If a drain is placed, how long does the drain remain in place?**	Up to 24 h	17/47 (36.2%)
	24–48 h	30/47 (63.8%)
	>48 h	(0%)
	Missing	6
**After burr‐hole craniostomy, do patients mobilize or do you prescribe bedrest?**	Bedrest as long as the drainage system is connected	32/48 (66.7%)
	To mobilize with an open drainage system	16/48 (33.3%)
	To mobilize with a closed drainage system	(0%)
	Missing	5
**Does operation room capacity play a role in the choice of primary CSDH treatment?**	No	38/49 (77.6%)
	Yes	11/49 (22.4%)
	Missing	4
**If operation room capacity plays a role in the choice of primary CSDH treatment, which treatment do you prefer until operation?**	Expectative policy until operation	5/11 (45.5%)
	Medical treatment until operation	6/11 (54.5%)
	*Dexamethasone*	*6/6 (100%)*
	*Tranexamic acid/other*	*(0%)*

The majority (83%) of responding Dutch neurosurgeons perform BHC under general anesthesia. When performing a BHC, one burr‐hole is chosen by 68%, with the side note that this depends on the size of the hematoma. If a bilateral CSDH occurs, most (70%) of responding neurosurgeons operate on both sides if technically possible.

The subdural space is flushed by almost all (96%) responding neurosurgeons; of whom 21% flushes on room temperature and 79% on body temperature. The irrigation fluid most commonly used (59%) is saline solution. The use of burr‐hole covers is found unnecessary by 56% of responding neurosurgeons; 33% would be open to the use of burr‐hole covers. A drain is placed by almost all (98%) responding neurosurgeons; 24% subgaleal and 76% subdural. Drains are most often (64%) left in situ for 24–48 h. Postoperative, 67% of patients have bedrest until removal of the drain. According to 78% of responding neurosurgeons, the capacity of operation rooms and operation time do not play a role in the choice of primary treatment.

## 4 DISCUSSION

Worldwide, but also on a national level, there is a broad variation in CSDH‐management. The aim of this survey was to make a baseline inventory of current clinical practice and current attitudes of Dutch neurologists and neurosurgeons toward the management of CSDH. Through this survey, we detected familiarity with possible treatments, with possible environmental barriers and with inertia of current practice. This is important because a lack of agreement with guideline recommendations, due to lack of applicability or lack of evidence, is the most perceived barrier to guideline adherence (Cabana et al., 1999; Lugtenberg et al., 2009). We found variation in current clinical practice and current attitudes among treating physicians. Since most respondents would be willing to adopt new treatments if positive effects are shown in RCTs, differences in current clinical practice and current attitude are considered not to be insurmountable when eventually implementing evidence‐based treatments. This baseline inventory is considered as one of the stepping‐stones in the eventual establishment and implementation of evidence‐based treatments.

CSDH is presented by a large variability of symptoms (Kolias et al., 2014). In this survey, we incorporated four fictitious patient cases to evaluate which treatment option was preferred by the respondents in a specific situation. The respondents’ answers to these cases represent the consensus and variability in treatment preferences in different situations. The cases described fictitious patients with mild to very severe symptoms having small to large hematomas on a CT scan. Most respondents (83%) chose to perform burr hole craniostomy in the fictitious patient from case 1, who was most severely affected neurologically with a large CSDH. Conservative treatment was chosen by a majority of respondents (65%) for the patient in case 2, who had no neurological symptoms at presentation with a small hematoma. Cases 3 and 4 both concerned a patient with an average‐sized CSDH, in both cases combined with the pronation and drift of an arm. In case 4, speech disturbance was additionally present and in this case, burr hole craniostomy was most often chosen (66%). In case 3, without the speech disturbance, the treatment choice was divided over the different treatment options. Burr‐hole craniostomy still was the preferred option (41%), but not chosen by the majority of respondents. These findings show that in severe or mild CSDH, most neurologists and neurosurgeons agree on the primary treatment, but in a moderately affected patient it may be more difficult to reach consensus. CSDH remains a complex disease in which a guideline would be beneficial on medical decision making.

The findings in the cases suggest BHC is the most widely used and most accepted treatment option in symptomatic CSDH, which is in line with up‐to‐date literature (Kolias et al., 2014; Soleman et al., 2017). A majority of respondents has a positive attitude toward a conservative policy and around a third had a positive experience with the use of dexamethasone as a primary and additional treatment. These numbers were also reflected in the treatment preferences in the presented cases. Ninety‐six percent of respondents did not have any experience with middle meningeal artery embolization. Although this procedure has been suggested as an adjuvant and/or alternative intervention for CSDH treatment with a reduced recurrence rate (Catapano et al., 2021; Onyinzo et al., 2021; Shotar et al., 2020), it is not commonly performed in Dutch hospitals as of today. (Peri)operative management largely corresponded among responding neurosurgeons. Remarkably, a majority of responding neurosurgeons prefer a subdural drain to a subperiosteal drain, despite the most recent publications presenting less recurrence when using a subperiosteal drain (Greuter et al., 2020; Pranata et al., 2020; Soleman et al., 2019). This is an illustrative example of the inertia in the change of practice. This inertia might be affected by a lack of familiarity with the use of subperiosteal drains. In addition, if CSDH‐guidelines were available, these could help in keeping clinical practice up‐to‐date.

This survey examined examples of environmental barriers, such as organizational restraints and collaborative challenges, and the experience with these possible barriers among the respondents. For example, in terms of organizational problems, no barriers were found in operation room capacity. Collaboration did not seem to be a barrier either. Neurologists seem to consult neurosurgeons without hesitation and these specialists work closely on optimal CSDH‐management in each patient. Thus, in this survey, no environmental barriers were found.

The strength of this study is that a clear baseline inventory on current clinical practice and current attitudes toward the management of CSDH in the Netherlands is now available. If countries attempt to establish and implement a national guideline on CSDH‐management, an insight into daily clinical practice and attitudes is a small but required element.

This survey has some limitations. First, the response rate of 8% (66/844) among Dutch neurologists is low compared to the response rate of 35% (53/153) among Dutch neurosurgeons. The actual neurological response rate might be higher because it is unknown how many of the 844 Dutch neurologists did see the invitation to fill in this survey on the website and LinkedIn page of the Dutch Neurology Association. Also, a neurologist, specialized in other areas of interest, might be less eager to fill in the questionnaire, which might lead to a moderate nonresponse bias (af Wåhlberg & Poom, 2015). The neurosurgical response rate is expected to be more reliable because all Dutch neurosurgeons received two emails from the Dutch Neurosurgical Society.

However, even if 844 neurologists did see the invitation, low response rates do not necessarily lead to less accurate measurements (Lindemann, 2019). The neurological response percentage is lower, but the accuracy in both groups matches and is acceptable.

A second limitation, which also concerns the response rate, is that, besides an online survey, not many other survey options were available in 2020 due to the COVID‐19 pandemic. We did plan on presenting this survey on national neurosurgical and neurological congresses, in order to increase the response rate and the interest in CSDH‐management. Unfortunately, this was not possible.

### 4.1 Future CSDH‐research

It is already known that the intraoperative placement of a subdural drain, remaining in situ up to 48 h, is associated with reduced recurrence and reduced mortality at 6 months (Santarius et al., 2009). Recently the first adequately sized multicenter RCT studying the role of dexamethasone in CSDH‐management was published (Hutchinson et al., 2020). In total, 680 patients were randomized for dexamethasone or placebo to test the hypothesis that dexamethasone would improve outcome by reducing the need for surgical intervention. The dexamethasone group had fewer favorable outcomes and more adverse events than placebo at 6 months, despite fewer reoperations. It should be noted that the large majority (94%) of patients in this study underwent surgery. Therefore, no definite conclusions could be drawn regarding the effect of dexamethasone as a method of conservative management to avoid surgery (Hutchinson et al., 2020). The DECSA trial will provide further insight on this matter (Miah et al., 2018).

The Danish Chronic Subdural Hematoma Study (DACSUHS), a national CSDH collaboration between four neurosurgical departments in Denmark, was established in order to standardize CSDH‐management and research within Denmark. DACSUHS has been the first to reach consensus on a CSDH guideline (Jensen et al., 2018), using the “Oxford Centre for Evidence‐Based Medicine: Levels of Evidence and Grades of Recommendation” to evaluate the evidence on CSDH and to support the recommendations (Centre for Evidence‐Based Medicine, 2009). The DACSUHS group chose 10 items. On each of these items, the DACSUHS members decided if there was a strong or weak recommendation for or against a particular treatment option. Consensus was reached and their recommendations were translated into a 10‐point national guideline (Table 4).

**TABLE 4:** Danish national guidelines on CSDH‐management (Rønn Jensen et al., 2018)

1	**When to operate?** To operate when the CSDH causes severe mass effect with or without neurological symptoms and signs. To offer conservative treatment to patients with mild and insignificant mass effect.
2	**Use of anticoagulants?** To revert antithrombotic treatment prior to surgery
3	**Type of surgery?** To use single burr hole as primary treatment
4	**Use of drain?** To use subdural drain after burr hole evacuation, not left in for longer than 24 h
5	**Type of drainage fluid?** To flush with isotonic fluid during evacuation of CSDH
6	**In bilateral CSDH?** To evacuate hematoma on both sides of bilateral CSDH
7	**Medical treatment?** Not to use adjuvant pharmacotherapy as part of the treatment of CSDH
8	**Bedrest or mobilization?** To offer elevation of headrest and early mobilization to patients after evacuation of CSDH
9	**Follow‐up CT?** To perform a CT head scan if the patient fails to recover after surgery, no control scan in asymptomatic patients
10	**Recurrent CSDH?** To consider craniotomy for recurrent CSDH

*Note*: Each item contains a question on CSDH‐management and the consensus reached on that particular item using the Oxford Centre for Evidence‐Based Medicine: Levels of Evidence and Grades of Recommendation (Centre for Evidence‐Based Medicine, 2009).

Following their example, we established the Dutch Subdural Hematoma Research group (DSHR) in 2018 (DSHR). The aim of the DSHR is to combine Dutch CSDH‐studies and eventually convert the results of (inter)national studies into a widely supported national guideline on the management of CSDH. This baseline inventory is a small but required part of the establishment and implementation of such a guideline.

Once sufficient (inter)national CSDH‐RCTs on a large variety of topics are completed (Edlmann et al., 2020), more robust evidence‐based decision‐making is possible. By that time, we are planning to conduct a national Delphi survey on the establishment and implementation of a national guideline, using this baseline inventory as a stepping‐stone in combination with the class I evidence from CSDH‐RCTs.

## 5 CONCLUSION

In this baseline inventory, we found variation regarding current practice and current attitudes toward CSDH‐management in the Netherlands. However, these differences are considered not to be insurmountable when implementing evidence‐based treatments. We advise all CSDH‐researchers to establish a national baseline inventory on current clinical practice and current attitudes toward CSDH‐treatment. This is a small but indispensable stepping‐stone in the eventual development and implementation of a national guideline.

## COLLABORATORS DUTCH SUBDURAL HEMATOMA RESEARCH GROUP (DSHR)

N. Asahaad, Van Weel Bethesda Ziekenhuis, Dirksland, The Netherlands; J. Boogaarts, Radboud UMC, Nijmegen, the Netherlands; C. de Brabander, Admiraal de Ruyter Ziekenhuis, Goes, the Netherlands; R.J.M. Groen, University Medical Center Groningen, Groningen, the Netherlands; R. Haeren, Maastricht University Medical Center, Maastricht, the Netherlands; S. Immenga, Amsterdam UMC, Amsterdam, the Netherlands; F. van Kooten, Erasmus MC, Rotterdam, the Netherlands; R. Kloppenborg, Haaglanden MC, The Hague, the Netherlands; R. Lodewijkx, Amsterdam UMC, Amsterdam, the Netherlands; W. Moudrous, Maasstad Ziekenhuis, Rotterdam, the Netherlands; W.C. Peul, University Neurosurgical Center Holland (UNCH), Leiden/The Hague, the Netherlands; G.J.J. Plas, Ziekenhuisgroep Twente, Almelo/Hengelo, the Netherlands; I.R. de Ridder, Maastricht University Medical Center, Maastricht, the Netherlands; A.D. Rozeman, Albert Schweitzer Hospital, Dordrecht, the Netherlands; Y. Temel, Maastricht University Medical Center Maastricht, the Netherlands; W.P. Vandertop, Amsterdam UMC, Amsterdam, the Netherlands; V. Volovici, Erasmus MC, Rotterdam, the Netherlands; D. de Waard, Sint Franciscus Gasthuis en Vlietland, Rotterdam, the Netherlands; A.D. Wijnhoud, IJsselland Ziekenhuis, Capelle aan den IJssel, the Netherlands

## CONFLICT OF INTEREST

The authors declare no conflict of interest.

### PEER REVIEW

The peer review history for this article is available at https://publons.com/publon/10.1002/brb3.2463

## Data Availability

The data that support the findings of this study are available from the corresponding author, D.C. Holl, upon reasonable request.
